# Chimeric cellobiohydrolase I expression, activity, and biochemical properties in three oleaginous yeast

**DOI:** 10.1186/s13068-020-01856-z

**Published:** 2021-01-06

**Authors:** Markus Alahuhta, Qi Xu, Eric P. Knoshaug, Wei Wang, Hui Wei, Antonella Amore, John O. Baker, Todd Vander Wall, Michael E. Himmel, Min Zhang

**Affiliations:** Biosciences Center, National Renewable Energy Laboratory, Golden, CO 80401 USA

**Keywords:** Metabolic engineering: chimeric protein, Oleaginous yeast, CBH I, Consolidated bioprocessing, Cellobiohydrolase, Cel7A

## Abstract

Consolidated bioprocessing using oleaginous yeast is a promising modality for the economic conversion of plant biomass to fuels and chemicals. However, yeast are not known to produce effective biomass degrading enzymes naturally and this trait is essential for efficient consolidated bioprocessing. We expressed a chimeric cellobiohydrolase I gene in three different oleaginous, industrially relevant yeast: *Yarrowia lipolytica, Lipomyces starkeyi,* and *Saccharomyces cerevisiae* to study the biochemical and catalytic properties and biomass deconstruction potential of these recombinant enzymes. Our results showed differences in glycosylation, surface charge, thermal and proteolytic stability, and efficacy of biomass digestion. *L. starkeyi* was shown to be an inferior active cellulase producer compared to both the *Y. lipolytica* and *S. cerevisiae* enzymes, whereas the cellulase expressed in *S. cerevisiae* displayed the lowest activity against dilute-acid-pretreated corn stover. Comparatively, the chimeric cellobiohydrolase I enzyme expressed in *Y. lipolytica* was found to have a lower extent of glycosylation, better protease stability, and higher activity against dilute-acid-pretreated corn stover.

## Background

Efficient deconstruction of lignocellulosic biomass for the purposes of the production of advanced biofuels is challenging. The natural recalcitrance of plant cell walls to degradation has led to several industrially relevant strategies to more efficiently produce monomeric, fermentable sugars from lignocellulosic biomass [1–4]. Fungal cellulase systems produce an array of non-peptide tethered glucoside hydrolases that act in concert to efficiently degrade lignocellulosic biomass [5, 6]. Consolidated bioprocessing (CBP) uses microorganisms that can break down the cellulosic and hemicellulosic fractions of pretreated biomass and simultaneously convert the released monomeric sugars to useful products [7–9]. Yeast can produce high yields of biofuels or their molecular precursors, making them a particularly interesting choice for CBP. For example, ethanol from *Saccharomyces cerevisiae* [10] and fatty acids or lipids from *Yarrowia lipolytica* [11, 12], *Lipomyces starkeyi* [13–15], and *S. cerevisiae* [16, 17]. However, these yeast are not naturally cellulolytic and must be engineered to secrete cellulase enzymes [1, 7].

To enable CBP capabilities in yeast, significant secretion levels of cellulases, particularly cellobiohydrolase I (CBH I) are needed [1, 18]. *S. cerevisiae* [7, 19–21], *Y. lipolytica* [22–24], *L. starkeyi* [25], or all three hosts [26] are often studied examples of yeast CBP organisms. Still, low secretion levels and yields of active enzymes have affected their usability [27–29]. To remedy this, different strategies have been attempted [30, 31]. For example, CBH I enzymes from different fungal species, including *Neurospora crassa,* are known to have performance properties similar to CBH I enzymes from *Trichoderma reesei* and have been screened and compared [22, 23]. Recently, *Y. lipolytica* has been engineered to degrade biomass allowing growth on Avicel [22, 23]. Furthermore, cell-surface expression of cellulases has shown promise [32, 33], and a tunable *S. cerevisiae* expression system has been shown to improve cellulolytic activity compared to a strain with three cellulase-expression constructs in a high-performance gene expression cassette [34].

Another strategy employed for fungal cellulase expression is making chimeric enzymes from domains of different fungal species that may be more easily secreted, such as the *Talaromyces emersonii* catalytic domain connected to the *T. reesei* linker peptide and carbohydrate binding module (CBM1) forming a chimeric *TeTr*CBH I [24]. Similarly, a fusion protein constructed with an easily secreted leading enzyme, endoglucanase II from *T. reesei*, followed by the more difficult to secrete *TeTr*CBH I was constructed and shown to be effective in improving cellulase secretion in *L. starkeyi* and *Y. lipolytica* [26]. Recently, this approach was taken further by introducing a translational fusion partner protein and screening for cellulases that were shown to have better secretion and improved ethanol production in *S. cerevisiae* [35].

The enzymatic properties of CBH I expressed in yeast are basically preserved, but the observed specific activity is lower than that of most other heterologous enzymes [27, 36], since yeast are known to struggle to properly fold the complete CBH I protein [21, 27, 30, 36–38]. Problems with proper disulfide bond formation and “hyper glycosylation” have been proposed to affect the yield of active CBH I [27, 30, 36, 37, 39, 40]. Although, there are some reports about characterization of heterologously expressed CBH I enzymes in a specific yeast host, most are limited to some individual property relevant to that specific host, and few of them have involved systematic analysis using various approaches across a diversity of yeast. A notable exception is our prior work comparing the endoglucanase II *TeTr*CBH I fusion construct in three yeasts [26]. While the goal of the fusion construct work was mainly aimed at understanding the benefits of the fusion, the results suggested significant *TeTr*CBH I expression and activity variations between yeasts. To properly understand the differences in just the CBH I part in yeast, we expressed a single chimeric *TeTr*CBH I gene in three oleaginous, industrially relevant yeast hosts: *Y. lipolytica, L. starkeyi,* and *S. cerevisiae*. We systematically analyzed their purified recombinant *TeTr*CBH I by various advanced approaches and characterized these enzymes in term of active enzyme recovery, glycosylation content, thermal and proteolytic stability, surface charge difference, as well as biomass deconstruction ability.

## Methods

### Cloning and expression

#### Gene cloning in *E. coli*

Gene cloning in *E. coli* DH5-alpha strain used standard gene cloning protocols [41], in which the Gibson Assembly Cloning Kit (NEB, Ipswich, MA, USA) was employed to insert target gene into expression vectors [25]. Oligos for PCR amplification typically consisted of 16-bp overlapping regions to facilitate Gibson based cloning followed by 20–26 bp of the region of interest.

#### Transformation

#### **L. starkeyi**

The chimeric *T. emersonii*–*T. reesei cbh1* (*TeTrcbh1*) gene was expressed in *L. starkeyi* driven by the *L. starkeyi* native pyruvate kinase (pyk) promoter detailed previously [25]. Wild-type (WT) *L. starkeyi* NRRL Y-11557 used as the transformation host was acquired from the ARS Culture Collection (NRRL) and was transformed as previously described [25, 42–43]. Briefly, a single colony of *L. starkeyi* was inoculated into YPD medium and incubated at 30 °C and shaken at 225 rpm until OD_600_ reached 8.0. Cells were harvested by centrifugation at 3000 rpm for 5 min, washed with 25 mL of water, and resuspended in 0.5 mL of 0.1 M LiAc. Suspended cells (100 µL) were dispensed into microcentrifuge tubes and briefly pulsed to collect the cells and remove the supernatant. The collected cells were resuspended in 240 µL of PEG (50% w/v, MW 3650), 30 µL of 1 M Li acetate, 3 µL of ssDNA (10 µg/µL), and linearized DNA in water to equal 27 µL (300 µL total volume), and vortexed thoroughly. Typically, 5 to 8 µg of linearized DNA was used per transformation. The mixture was incubated at 30 °C for 3 h without shaking and then heat treated at 40 °C for 5 min. The cells were pelleted by centrifugation for 15 s and the supernatant was removed. The cell pellet was resuspended in 1 mL of yeast extract-peptone-dextrose (YPD) medium and incubated at 30 °C and 225 rpm for 3 h. Cells were then centrifuged, resuspended in 0.5 mL of water, and plated on YPD with 30 µg/mL of nourseothricin (ClonNAT).

### Y. lipolytica

*Y. lipolytica* Po1g (MatA, leu2-270, ura3-302:URA3, xpr2-332, axp-2) and the secretion vector pYLSC1 were purchased from Yeastern Biotech Co. (Taipei, Taiwan). The vector (pYLSC1, 7205 bp) contains the hybrid promoter (hp4d) and a secretion signal (XPR2 pre-region): atgaagctcgctaccgcctttactattctcacggccgttctggcc, which encodes the signal peptide MKLATAFTILTAVLA. This vector also contains a leucine selection marker gene (*LEU2*), which can complement the deletion of *LEU2* gene in the parental strain of Po1g. The constructs were built in the backbone of the secretion vector, pYLSC1, using the hp4d promoter. The gene coding sequence of *TeTr*CBH I was codon-optimized based on the codon bias of *Y. lipolytica*, and was synthesized by GenScript (described in details previously [24]). *Y. lipolytica* was typically grown in YPD medium.

### S. cerevisiae

*S. cerevisiae* was grown in YPD for general growth and transformation. Transformants were grown in YNB media without uracil and containing glucose as the carbon source. *S. cerevisiae* was grown at 30 °C in shake flasks and shaken at 220 rpm. The CBH I expression plasmid for *S. cerevisiae* was built on pD1214 using a strong constitutive TEF promoter, CYC terminator, and URA3 for selection (https://www.atum.bio/products/expression-vectors/yeast). The *T. reesei* XYN 2 secretion signal peptide was added to the *TeTr*CBH I sequence to create an expression cassette for *S. cerevisiae* (Additional file 1). This plasmid, pSc35, was transformed into BFY716 (*S. cerevisiae* D5A *ura3*::APH 3′ II, *ura3*::HPH).

Transformant colonies were assayed for activity using a *p*NP-lactose activity assay (described below). The colony showing the most activity was selected for scale up in a 10-L fermenter.

### Fermentation

Production of *TeTr*CBH I was carried out using 10 L of growth media in a 14-L BioFlo 310 bioreactor (New Brunswick Scientific – Eppendorf, Edison, NJ). For *L. starkeyi* and *Y. lipolytica*, seed cultures were inoculated from a single colony into 50 mL of YPD medium in a 250 mL flask, incubated at 30 °C and shaken at 225 rpm. The cultures were then transferred after 24 h of incubation into 1 L of fresh YPD pH 5.0 medium in a 2.8 L baffled flask. The secondary seed culture was subsequently transferred into 10 L of medium in the 14-L BioFlo 310 bioreactor after ~ 36 h of incubation. The *L. starkeyi* fermentations were controlled at 30 °C, 300 rpm stirring, one volume of air per volume of media per minute (VVM) at pH 5.2 in YPD medium consisting of individually made components and 5% total glucose. The fermentation was run until OD_600_ reached a maximum value, which was usually between 72 and 96 h. *Y. lipolytica* fermentations were controlled at pH 5.0, 28 °C, 300 rpm, and one VVM air in YPD media. Media consisted of individually made components and included citric acid and sodium citrate for increased buffering. The fermentation was run until OD_600_ reached maximum, usually between 72 and 120 h. *S. cerevisiae* fermentations were controlled at pH 5.0, 30 °C, 300 rpm, one VVM air in YNB—ura medium with 5% total glucose. This fermentation was run for ~ 24 h, at which time all glucose was consumed. All culture broths were pelletized via centrifugation and concentrated using Tangential Flow Filtration (TFF) with a 10,000 MWCO membrane. The concentrated culture broths were buffer exchanged into 20 mM Bis–Tris pH 6.5 in preparation for chromatographic purification.

### Purification

After concentration and buffer exchange, the samples were further purified by chromatography. First the ammonium sulfate concentration of the sample was slowly adjusted to 1.5 M and filtered with a 0.45-μm Nalgene Rapid-Flow Bottle Top filter (Thermo Scientific Pierce Protein Biology Products, Rockford, IL, USA). Then the eluate was applied to a GE XK 26 column packed with hydrophobic interaction chromatography resin (Phenyl Sepharose 6 Fast Flow) and equilibrated with a 50 mM Bis–Tris pH 6.5 buffer containing 1.5 M ammonium sulfate. The partially purified sample was eluted from the column with a descending ammonium sulfate gradient and desalted in 20 mM Bis–Tris pH 6.5 buffer using two GE HiPrep 26/10 desalting columns connected in series. Next, the sample was applied to anion exchange chromatography using a Tricorn 10/100 column packed with GE Source 15Q resin in 20 mM Bis–Tris pH 6.5 buffer and an increasing NaCl gradient. Final purification was done with size exclusion chromatography using a GE 26/60 Superdex 75 column eluted with 20 mM acetate pH 5.0 buffer containing 100 mM NaCl. Whenever necessary, Vivaspin 20 10 kDa concentrators were used to concentrate the samples. The desired protein fractions were identified using the *p*-nitrophenyl-β-lactoside assay [44]. All chromatography columns, resins, and concentrators were purchased from GE Healthcare (Piscataway, NJ, USA). Protein purity was assessed by SDS-PAGE and concentration was determined using A_280_. The values given in Table 1 are from one purification batch. *Y. lipolytica* and *L. starkeyi TeTr*CBH I enzymes were purified several times with similar results. Only one purification batch was needed for *S. cerevisiae TeTr*CBH I. *T. reesei* CBH I (*Tr*CBH I) was cloned, expressed, and purified as described before [24].

**Table 1:** Yield of purified and active *TeTr*CBH I expressed in *Y. lipolvtica*, *L. starkeyi,* and *S. cerevisiae*

Protein	Protein yield (mg/L)
*TeTr*CBH I in *Y. lipolvtica*	1.1
*TeTr*CBH I in *L. starkeyi*	0.1
*TeTr*CBH I in *S. cerevisiae* peak 1	0.8
*TeTr*CBH I in * S. cerevisiae* peak 2A	1.1
*TeTr*CBH I in * S. cerevisiae* peak 2B	1.1
*TeTr*CBH I in * S. cerevisiae* (total of three isoforms)	3.0

### High performance liquid chromatography

To verify the purity of the chimeric CBH I enzymes, HPSEC with TSK gel G3000SWXL column (Tosoh Bioscience, Tokyo, Japan) was used. Both UV (at 280 nm; Rainin Dynamax UV-D II) and RI (Jasco, A029660872) detectors were used to obtain information about glycosylation differences. One hundred µL of approximately 2 mg/mL protein sample were loaded onto the HPSEC column at a flow rate of 0.5 mL/min in 20 mM acetic acid pH 5 with 100 mM NaCl.

### Proteolytic stability

Protein samples at a concentration of 0.25 mg/mL were incubated with Thermolysin (Promega, Madison, USA) or α-chymotrypsin (Promega, Madison, USA) at 25 μg/mL or 4 μg/mL, respectively, in a volume of 200 μL for 0.5 h, 1 h, 2 h, and 4 h. Thermolysin and α-chymotrypsin reactions were stopped by addition of 1% EDTA (Sigma-Aldrich, St. Louis, USA) and a protease inhibitor cocktail (Sigma-Aldrich, St. Louis, USA), respectively. All samples, including the time zero (t_0_) point were loaded on a NuPAGE Bis–Tris Gel (Thermo Fisher Scientific Inc., Rockford, USA) for electrophoresis and densitometry analyses, performed with Image J [45].

### Thermal stability

Thermal stability was measured using a Microcal model VP-DSC calorimeter (Microcal, Inc., Northampton, USA). Data analysis was completed by Origin for DSC software (OriginLab, Northampton, USA). Samples were diluted up to 100 μg/mL protein in 20 mM acetate pH 5.0 buffer with 100 mM NaCl. The calorimeter scan rate was 60 °C/h over a range of 25–80 °C.

### Comparative SDS-PAGE

Protein samples were diluted in 4 × LDS sample buffer (Life Technologies Corp., Carlsbad, USA) and incubated at 95 °C for 10 min prior to loading on a NuPAGE Bis–Tris Gel (Thermo Fisher Scientific Inc., Rockford, USA). Electrophoresis was conducted in MOPS (3-(N-morpholino) propanesulfonic acid) buffer and 200 V for 50 min. Coomassie staining was performed using Thermo Scientific™ Pierce™ Power Stainer (Thermo Fisher Scientific Inc., Rockford, USA), whereas staining for glycosylation was done using the Pierce™ Glycoprotein Staining Kit (Thermo Fisher Scientific Inc., Rockford, USA), following the manufacturer’s instructions. Western-Blot analyses were performed as reported earlier [46].

### Isoelectric focusing

A Novex pH 3–7 IEF Protein Gel system (Invitrogen, NY) was used for isoelectric focusing (IEF) experiments using manufacturer recommended protocols and IEF Markers pH 3–10 (Invitrogen, NY). Approximately 80 µg of protein concentrate was loaded into each well.

### Digestion assays

Cellulase activity was measured using dilute-acid-pretreated corn stover (PCS) as a substrate. Specifically, the PCS used was NREL dilute-acid-pretreated corn-stover P050921, produced in a vertical pulp digester supplied by Sunds Defibrator (now Metso Corporation, Helsinki, Finland) as described earlier [47], with a residence time of approximately one min at 190 °C and 0.45 g H_2_SO_4_ per g dry biomass at 30% solids loading. These conditions yielded a material having 59.1% glucan, 5.1% xylan, and 25.3% lignin. Because the theoretical molecular weight of the *TeTr*CBH I is slightly greater than that of the *Tr*CBH I, an equal molar loading resulted in a loading of 25.0 mg enzyme/g cellulose for *TeTr*CBH I, versus 24.6 mg enzyme/g cellulose for *Tr*CBH I. This equals a loading of 5.7 mg enzyme/mL PCS. In addition to purified *TeTr*CBH I enzymes, each assay also contained the catalytic domain of *Acidothermus cellulolyticus* E1 endoglucanase (with the enhanced Y245G mutation [48]) loaded at 1.7 mg enzyme/g cellulose, along with *Aspergillus niger* beta-glucosidase (BGL). BGL was chromatographically purified from the commercial mixture Novozyme 188 (Novozymes North America, Franklinton, NC, USA) and was loaded into the reaction mixtures at a concentration of 0.4 mg enzyme/g of cellulose substrate.

Assays were carried out at 40 °C in 20 mM acetate pH 5.0 buffer containing 0.02% (w/v) sodium azide to inhibit microbial growth. Assays were performed in triplicate using initial digestion volumes of 1.7 mL in crimp-sealed 2.0-mL HPLC vials which were constantly mixed by inversion (10 revolutions per min) in a 40 °C water bath. At designated time points during the digestions, representative 0.1-mL aliquots of liquid and solids were withdrawn for analysis. These aliquots were diluted 18-fold with deionized water into sealed 2.0-mL HPLC vials and immersed for 10 min in a boiling water bath to terminate the enzymatic reactions. The diluted aliquots were then filtered through 0.2-μm filters before determination of released sugars by HPLC, as described previously [24].

## Results and discussion

The *TeTrcbh1* fusion gene was expressed in *Y. lipolytica, L. starkeyi,* and *S. cerevisiae*. The recombinant *TeTr*CBH I enzymes were purified and used for further characterization. Our prior work with the CBH I-endoglucanase II fusion protein showed variability in the active protein expression levels and in biomass deconstruction efficacy when expressed in different yeast species [26]. To better understand the basis of these differences in CBH I performance, without interference from the fusion partner, we planned a series of experiments investigating the yields of purified and active *TeTr*CBH I enzyme, sample heterogeneity (e.g., glycosylation and charge differences), thermal and proteolytic stability, and performance. These experiments were performed with *TeTr*CBH I expressed in *Y. lipolytica, L. starkeyi,* and *S. cerevisiae* to better understand the behavior of this fusion construct across relevant oleaginous yeast species.

### Identification and recovery of *TeTr*CBH I expressed in *Y. lipolytica, L. starkeyi,* and *S. cerevisiae*

**Fig. 1:**
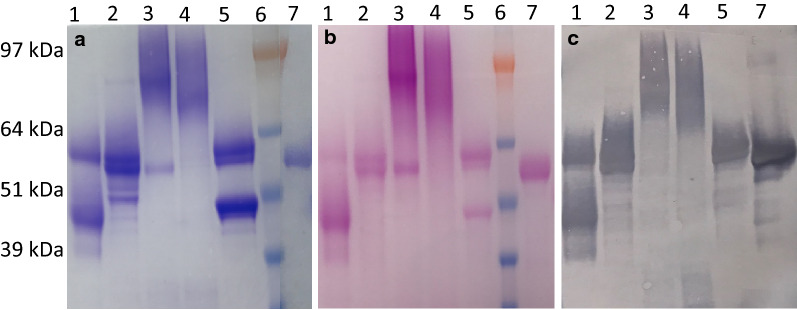
Comparative SDS-PAGE. **a** Coomassie stain, **b** Pierce™ Glycoprotein Stain, and **c** Western blot. Lanes: 1. *TeTr*CBH I expressed in *Y. lipolvtica*; 2. *TeTr*CBH I expressed in *L. starkeyi*; 3. *TeTr*CBH I expressed in *S. cerevisiae* peak 1; 4. *TeTr*CBH I expressed in *S. cerevisiae* peak 2A; 5. *TeTr*CBH I expressed in *S. cerevisiae* peak 2B; 6. MW standard, and 7. *Tr*CBH I control

The purified recombinant proteins from the three yeast species were identified by SDS-PAGE and Western blot (Fig. 1). First, we compared the recovery of purified and active *TeTr*CBH I from *Y. lipolytica, L. starkeyi,* and *S. cerevisiae* (Table 1). By only collecting fractions displaying activity on *p*NP-lactose [27], the purity of the enzymes studied was enhanced and this assured that the proteins we purify were active. *L. starkeyi* cultures had the lowest recovery of purified and active *TeTr*CBH I (0.1 mg/L), followed by *Y. lipolytica* (1.1 mg/L), representing a greater than tenfold increase in titer. The low recovery of active enzyme from *L. starkeyi* agrees with our earlier work with the CBH I-endoglucanase II fusion protein [26], where the fusion construct was necessary to improve the *L. starkeyi TeTr*CBH I yield. The *S. cerevisiae* enzyme was recovered at an even higher yield (3.0 mg/L), which is more than a 30-fold increase*.* These results are generally consistent with previously reported enzyme yield values [37, 39, 49]. However, the recovery of 1.1 mg enzyme/L for *Y. lipolytica* is much lower than previously reported, which is based on total secreted protein yield (i.e., 24 mg/L) [24]. The total secreted protein yield reported by this earlier study was based on Western blot densitometry and likely includes partially degraded, misfolded, incorrectly modified, and/or inactive enzymes. These data are further examples that demonstrate the low yields of active CBH I that are typically purified from yeast. Heterogeneity from possible misfolding, proteolysis, and modification appears to be considerable when *TeTr*CBH I is expressed in *S. cerevisiae,* where the production level is almost threefold higher than in *Y. lipolytica*. However, in this case there were three distinct and active *TeTr*CBH I isoforms (Additional file 2). The different isoforms first appeared during anion exchange chromatography indicating possible surface charge differences and were further separated by SEC. Closer inspection of the SEC peaks showed that all three peaks were indeed of slightly different size (Fig. 1 and Additional file 2). This finding strongly suggests that *S. cerevisiae* expresses active *TeTr*CBH I variants which differ in surface charge and size. These results can be explained by folding issues, but given that the enzymes are active, variability in glycosylation is also indicated. Glycan decoration of proteins impacts the surface charge and can significantly alter the protein size.

### Extent of glycosylation and heterogeneity

**Fig. 2:**
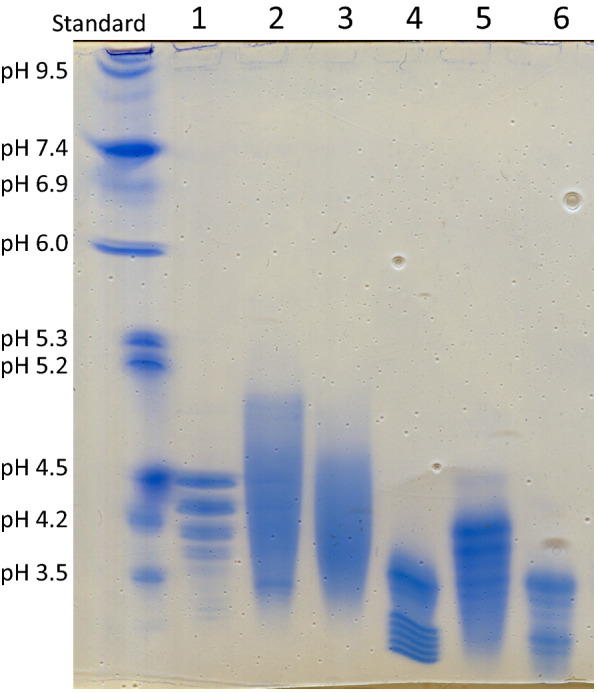
Isoelectric focusing gel. Lanes: 1. *Tr*CBH I; 2. *TeTr*CBH I expressed in *S. cerevisiae* peak 1; 3. *TeTr*CBH I expressed in *S. cerevisiae* peak 2A; 4. *TeTr*CBH I expressed in *S. cerevisiae* peak 2B; 5. *TeTr*CBH I expressed in* Y. lipolvtica*; 6. *TeTr*CBH I expressed in *L. starkeyi*. Standards: cytochrome C (10.7 pI), ribonuclease A (9.5 pI), lectin (8.3, 8.0, 7.8 pI), myoglobin (7.4, 6.9 pI), carbonic anhydrase (6.0 pI), β-lactoglobulin (5.3, 5.2 pI), trypsin inhibitor (4.5 pI), glucose oxidase (4.2 pI), amyloglucosidase (3.5 pI)

To assess the heterogeneity and the extent of glycosylation of the chimeric *TeTr*CBH I enzymes produced in the three yeast, several methods were used including HPSEC (Table 2), SDS-PAGE with Coomassie blue staining (Fig. 1a), glycosylation staining (Fig. 1b), and Western blot analysis (Fig. 1c), and isoelectric focusing (Fig. 2). All of the *TeTr*CBH I samples tested showed multiple apparent MW bands in the Western blot and glycosylation stained PAGE, indicating degradation and/or variable glycosylation extent. Curiously, some of the predominant bands were of low apparent molecular weight. This is especially true for the *TeTr*CBH I expressed in *Y. lipolytica*. To verify and quantify all sample components, we analyzed them with HPSEC and compared the results to *Tr*CBH I expressed in *T.* reesei (Table 2, HPSEC graphs in Additional file 2).

**Table 2:** HPSEC peak mobility and RI/UV ratios. Higher numbers for the RI/UV area ratio indicate higher glucan content. HPSEC graphs in Additional file 2

	RI peaks mobility (minutes)	RI combined peak area	UV peaks mobility (minutes)	UV combined peak area	RI/UV area ratio
*Tr*CBH I	15.12, 18.68	1202	14.95	2638	0.46
*TeTr*CBH I in *S. cerevisiae* peak 1	13.30, 20.42	1625	13.25	2424	0.67
*TeTr*CBH I in *S. cerevisiae* peak 2A	13.10, 19.42	1487	13.08	2260	0.66
*TeTr*CBH I in *S. cerevisiae* peak 2B	18.23, 21.20	1130	17.33, 18.57	2848	0.40
*TeTr*CBH I in *Y. lipolytica*	15.07, 19.97	1130	14.92, 18.45, 21.18	2639	0.43
*TeTr*CBH I in *L. starkeyi*	15.18, 19.20	1525	15.15, 21.23	2486	0.61

### Sample purity, degradation, and glycosylation

The HPSEC results agreed with the SDS-PAGE analysis and showed multiple peaks (Table 2, HPSEC graphs in Additional file 2) for *TeTr*CBH I expressed in each yeast species. Furthermore, HPLC with both RI and UV detectors allowed reliable measurements of the contents of each sample. The main peaks for all samples, except peak 2B of *TeTr*CBH I expressed in *S. cerevisiae*, had similar retention times to *Tr*CBH I; with only minor or insignificant secondary peaks (Fig. 1a, c). Degradation can be expected to show up as separate peaks in chromatography and as distinct bands in SDS-PAGE, whereas variable glycosylation results in broad peaks and smeared bands. *S. cerevisiae TeTr*CBH I peak 2B had two major peaks with significantly longer retention values and two “sharp” bands in SDS-PAGE, indicating significant degradation and low extents of glycosylation. The other two *S. cerevisiae TeTr*CBH I samples, peak 1 and peak 2A, eluted as single peaks, but were of much higher apparent molecular weight and appeared as elongated and smeared SDS-PAGE bands, suggesting a high glycan content.

### Western blot and sample degradation

The Western blot results (Fig. 1c) mostly agreed with the Coomassie stained PAGE (Fig. 1a), showing only minor bands that were not visible in Western blot. This result is expected, because our purification protocol selects for active fractions. Inactive fractions can only be fractionated if they co-elute with active forms of the enzyme. *S. cerevisiae* peak 1 and peak 2B were the most notable exceptions. Both fractions had low or lower molecular weight bands that were barely visible. It is possible that these species are unrelated impurities; however, the *S. cerevisiae* peak 1 is more likely to be a low concentration contamination from peak 2B that was not fully separated during purification. In the case of enzymes expressed from all yeast hosts studied, Western blot stained bands can be observed which verifies CBH I content. If degraded forms of CBH I have a weaker Western blot signal, both species can be assumed to be CBH I. This could be the case especially if a significant region, such as the CBM1 domain (and the linker peptide), is lost. The lower molecular weight SDS-PAGE bands of *S. cerevisiae* peak 2B and *Y. lipolyctica* could reflect this outcome (Fig. 1). Overall, the SDS-PAGE results for yeast expressed *TeTr*CBH I in Fig. 1 show potential hyper-glycosylation, degradation, and double bands compared to the *T. reesei* expressed *Tr*CBH I, again demonstrating the problems yeast have with CBH I expression and stability.

### Glycosylation

Using both UV and RI detectors in HPSEC allowed us to quantify the extent of glycosylation as an RI/UV peak area ratio due to the insignificant absorption of glycosylation at 280 nm, whereas it is readily detectable by the RI-detector. Higher numbers for the RI/UV area ratio indicate a higher glycan content. As expected from our previous results*, S. cerevisiae TeTr*CBH I peak 2B and *Y. lipolytica TeTr*CBH I have a lower glycan content (Table 2). Also, *S. cerevisiae TeTr*CBH I peak 2B has clear signs of degradation (two major peaks), whereas *Y. lipolyctica TeTr*CBH I shows two minor peaks in HPSEC. In contrast, *T. reesei Tr*CBH I migrates as a single band in SDS-PAGE (Fig. 1a). Clearly, *T. reesei* expression produces intact *Tr*CBH I with a relatively low extent of glycosylation, which contrasts with yeast which secrete a highly glycosylated *TeTr*CBH I. The RI/UV ratio of *S. cerevisiae TeTr*CBH I peaks 1 and 2A show a wide range of glycosylation extent, which agrees well with the glycan stained PAGE shown in Fig. 1b. Both species show no bands below the molecular weight of CBH I expressed in *T. reesei*, suggesting that glycosylation might protect the enzyme from degradation. This is clearly demonstrated in the case of *Y. lipolyctica TeTr*CBH I, which appears to be less glycosylated by PAGE analysis than the *L. starkeyi TeTr*CBH I and on Western Blot migrates as a main band below 50 kDa (Fig. 1c). These results strongly suggest that *Y. lipolyctica TeTr*CBH I is somewhat degraded. If the difference in molecular weight relates to the loss of the CBM1 and linker regions, this result could indicate that *Y. lipolyctica* has difficulty in adequately *O*-glycosylating the linker peptide region which is consequently degraded.

### Surface charge differences

To further characterize the physical differences between these enzymes, we performed isoelectric focusing (IEF, Fig. 2). IEF produced a unique isoelectric point distribution for each enzyme from the three yeast species tested. The results agreed with the overall extent of glycosylation. Interestingly, both *S. cerevisiae TeTr*CBH I peak 2B and *L. starkeyi TeTr*CBH I had isoelectric point distributions at a particularly low pH. Overall, these results show a significant variability of surface charge which correlates to the difference in glycosylation extent among the samples. Enzymes produced by these two yeast clearly have different glycosylation content compared to the other yeast expression products and *Tr*CBH I, perhaps a consequence of the differences in their protein glycosylation pathways. This concept has been widely reported in literature, particularly in relation to the variability in the extent and complexity of N-glycosylation of secreted proteins [50].

### Thermal and proteolytic stability

Glycosylation has been reported to strongly affect cellulase stability [51]. To understand the potential effects of glycosylation on performance, we performed thermal and proteolytic stability experiments comparing the *TeTr*CBH I produced in yeast to *Tr*CBH I expressed in *T. reesei*. All the yeast-expressed *TeTr*CBH I chimeras, except that expressed in *Y. lipolytica*, had a similar thermal stability with a *T*_max_ close to 65 °C, which is the value measured for *Tr*CBH I (Table 3). A significantly lower *T*_max_ (61.8 °C) was measured for *Y. Lipolytica TeTr*CBH I. This result is consistent with the notion that glycosylation, and especially *O*-glycosylation, is often correlated to thermal stability of cellulases and other proteins [52–54]. When considered together, the increased degradation (Fig. 1), low extent of glycosylation, and unique surface charge properties (Fig. 2) of *Y. lipolytica TeTr*CBH I suggest that the glycosylation of these proteins differ when compared to the native enzyme and that this could be the reason for the lower thermal stability shown by the heterologous enzyme.

**Table 3:** Thermal stability of *Tr*CBH I purified from *T. reesei* and *TeTr*CBH I purified from three yeast hosts

Protein	T_max_ (˚C)
*Tr*CBH I	65.1 ± 0.9
*TeTr*CBH I in *Y. lipolytica*	61.8 ± 0.7
*TeTr*CBH I in *L. starkeyi*	64.9 ± 0.2
*TeTr*CBH I in *S. cerevisiae* peak 1	65.7 ± 0.3
*TeTr*CBH I in *S. cerevisiae* peak 2A	65.6 ± 0.3
*TeTr*CBH I in* S. cerevisiae* peak 2B	64.9 ± 0.5

**Fig. 3:**
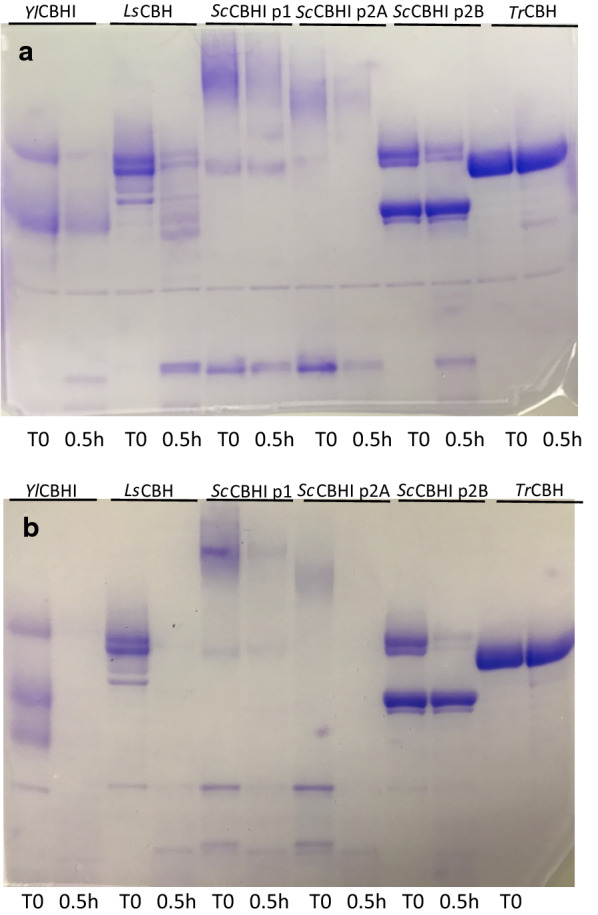
**a** Thermolysin treatment. T0: initial sample before incubation. 0.5 h: Sample after 0.5-h incubation. **b** α-chymotrypsin treatment. T0: initial sample before incubation. 0.5 h: Sample after 0.5-h incubation

Ttreatment with thermolysin (Fig. 3a) and α-chymotrypsin (Fig. 3b) showed very little degradation for *Tr*CBH I, whereas all yeast-expressed *TeTr*CBH I enzymes showed significant loss of protein. Interestingly, the lower molecular weight peak of *S. cerevisiae* peak 2B was not affected by proteases and was of similar size as the *Y. lipolytica* and *L. starkeyi TeTr*CBH I enzymes after proteolysis treatment. This species, therefore, is likely a degraded form of the CBH I catalytic module that cannot be further cleaved by these enzymes. Clearly, yeast expressed *TeTr*CBH I has lower proteolysis stability, supporting previous results, where CBH I expression is enhanced by expressing them in host yeast with protease gene deletions.

### Biomass deconstruction assays

**Fig. 4:**
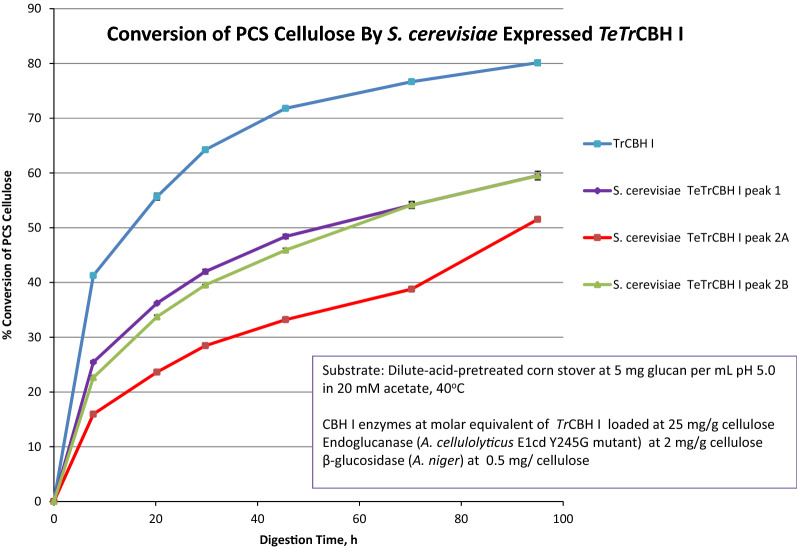
Conversion of PCS cellulose by *TeTr*CBH I enzymes purified from *S. cerevisiae* and *T. reesei*

The three purified chromatographic forms of *S. cerevisiae* expressed *TeTr*CBH I were evaluated for PCS cellulose degradation and compared to *Tr*CBH I purified from its native host, *T. reesei* (Fig. 4). All three forms of the *S. cerevisiae TeTr*CBH I showed a significantly lower extent of cellulose conversion compared to *Tr*CBH I. Surprisingly, peak 2B performed similarly to peak 1 despite being degraded into two peaks according to HPLSEC analysis (Additional file 2: Fig. S5). In contrast, peak 2A performed markedly worse. Thus, in this case, the degradation of peak 2B does not appear to have negatively impacted activity, highlighting the different performance of the various fractions derived from the same enzyme.

**Fig. 5:**
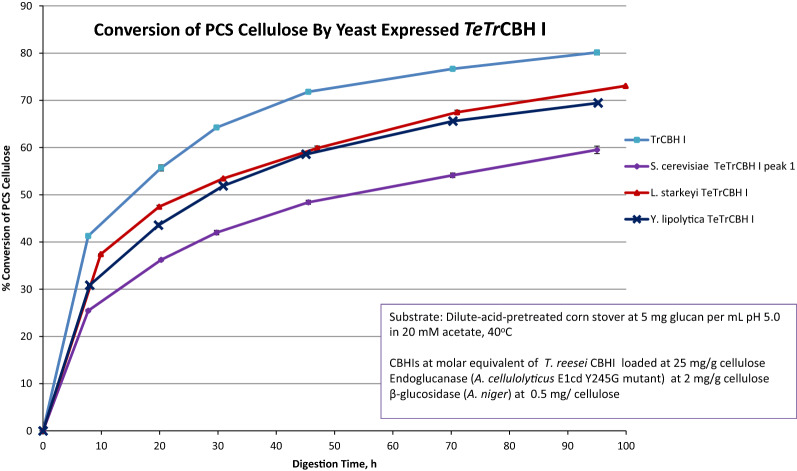
Conversion of PCS cellulose by *TeTr*CBH I enzymes purified from *S. cerevisiae, L. starkeyi, Y. lipolytica,* and *T. reesei*

The chimeric *TeTr*CBH I purified from *L. starkeyi* and *Y. lipolytica* converted 70% of the available PCS cellulose compared to 80% conversion for the *Tr*CBH I purified from its native host (Fig. 5). Note that peak 1 from *S. cerevisiae* converted only 60% of the substrate. Despite taking the best fraction for *S. cerevisiae* expressed *TeTr*CBH I, it still underperformed compared to the *L. starkeyi* and *Y. lipolytica* expressed chimeric *TeTr*CBH I. However, *Tr*CBH I clearly is more active than the *L. starkeyi* and *Y. lipolytica* expressed *TeTr*CBH I chimeras shown by reaching a conversion of over 80% in 100 h, compared to about 70% for the other two enzymes. These results show that there are significant *TeTr*CBH I activity differences between the three yeast, and that *Y. lipolytica* is the best host for stability and activity.

Our results agree with previous observations of lower CBH I enzyme activity when expressed in yeast [27, 36]. However, the significant activity differences between chimeric *TeTr*CBH I fractions isolated from one *S. cerevisiae* expression batch is new. These not only enrich our knowledge of the CBH I activity diversity expressed in yeast, but also reveals the complexities of CBH I expression in yeast. Our results show that the causes of this variability can be from differences in the genetic background of the yeast hosts which leads to folding, glycosylation, and proteolytic stability differences.

While yields and stability of heterologously expressed enzymes are important characteristics, it is crucial that the enzymes produced are sufficiently active to perform the expected function. For CBP, the hydrolysis of cellulose at a rate sufficient to permit cell growth is essential. Our study provides fundamental information about chimeric *TeTr*CBH I expressed in different yeast through systematical analysis. This deeper understanding is necessary to enable enhanced CBH I expression in yeast, thus helping to create new CBP yeast with efficient lignocellulose degradation capability.

## Conclusions

We report studies of the heterologous expression, biochemical and catalytic properties, and biomass deconstruction potential of chimeric *TeTr*CBH I enzymes expressed in three different, oleaginous, industrially relevant yeast: *Y. lipolytica, L. starkeyi,* and *S. cerevisiae*. Yields of purified active *TeTr*CBH I, sample heterogeneity (glycosylation and charge differences), thermal and proteolytic stability, and performance were determined to better understand cellulase expression in yeast. We show that *S. cerevisiae* demonstrates the highest yield of active *TeTr*CBH I construct; however, this enzyme appears to be the lowest in performance capability on a biomass substrate (Fig. 5). The *L. starkeyi* and *Y. lipolytica* expressed chimeric *TeTr*CBH I enzymes have comparable activity, which is also much higher than that of the *S. cerevisiae* produced enzyme. *Y. lipolytica* produces over ten times more purified and active *TeTr*CBH I enzyme than *L. starkeyi* (Table 1). Clearly, *L. starkeyi* and *S. cerevisiae* are inferior CBP candidates compared to *Y. lipolytica*. Based on these results, *Y. lipolytica* could, therefore, perform adequately in CBP from the perspective of producing sufficiently active CBH I; however, the available enzyme titers may not be high enough to function in an industrial setting [22, 23].

## Supplementary Information

**Additional file 1.** Schematic representation of the *TeTrCBH* I expression construct in *S. cerevisiae.*

**Additional file 2.** High performance size exclusion chromatography UV and RI chromatograms.

## Data Availability

The datasets used and/or analyzed during the current study are available from the corresponding author on reasonable request. All data generated or analyzed during this study are included in this manuscript and additional files.

## Abbreviations

CBP: Consolidated bioprocessing
T. reesei: Trichoderma reesei
S. cerevisiae: Saccharomyces cerevisiae
Y. lipolytica: Yarrowia lipolytica
L. starkeyi: Lipomyces starkeyi
CBH I: Cellobiohydrolase I
TrCBH I: T. reesei CBH I
TrEG II: T. reesei EG II
TeTrCBH I: A chimeric CBH I generated by the fusion of the catalytic module from Talaromyces emersonii CBH I with the linker peptide and cellulose-binding module from T. reesei CBH I
VVM: Volume of gas per volume of media per minute
PCS: Dilute-acid-pretreated corn stover
HPSEC: High-performance size exclusion chromatography
SDS-PAGE: Sodium dodecyl sulfate–polyacrylamide gel electrophoresis
