# Supplementary material for: Chimeric cellobiohydrolase I expression, activity, and biochemical properties in three oleaginous yeast
Source: Biotechnol Biofuels. 2021 Jan 6;14:6. doi: 10.1186/s13068-020-01856-z (PMC7789491; doi:10.1186/s13068-020-01856-z)
Supplement: Supplementary file 1 — Additional file 1. Schematic representation of the TeTrCBH I expression construct in S. cerevisiae. [file 13068_2020_1856_MOESM1_ESM.docx]

####
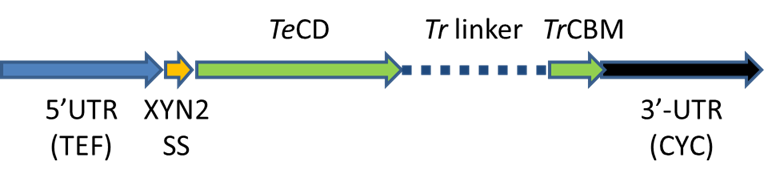


**Additional figure 1.** Expression construct for the *TeTr*CBH I in *S. cerevisiae*. Blue arrow: *S. cerevisiae* TEF promoter. Yellow arrow: *T. reesei* XYN 2 secretion signal peptide. Short green arrow and blue dashed line: *T. reesei* CBH I CBM and linker (*Tr*CBM + linker). Long green arrow: *T. emersonii* CBH I catalytic domain (*Te*CD). Black arrow: *S. cerevisiae* CYC terminator.
