# Supplementary material for: Chimeric cellobiohydrolase I expression, activity, and biochemical properties in three oleaginous yeast
Source: Biotechnol Biofuels. 2021 Jan 6;14:6. doi: 10.1186/s13068-020-01856-z (PMC7789491; doi:10.1186/s13068-020-01856-z)
Supplement: Supplementary file 2 — Additional file 2. High performance size exclusion chromatography UV and RI chromatograms. [file 13068_2020_1856_MOESM2_ESM.docx]

## HPLC to verify protein purity


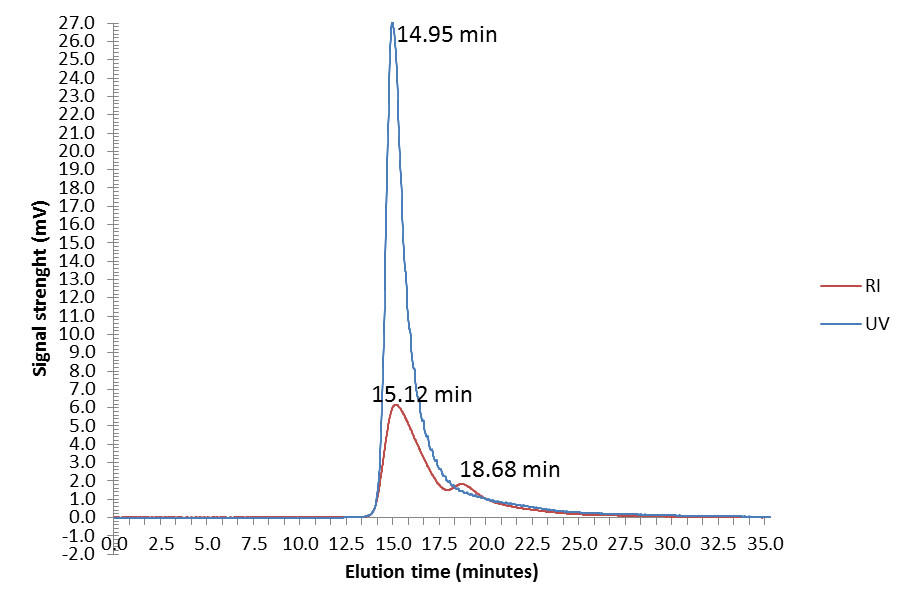


Additional figure 2. *Tr*CBHI HPSEC graph.


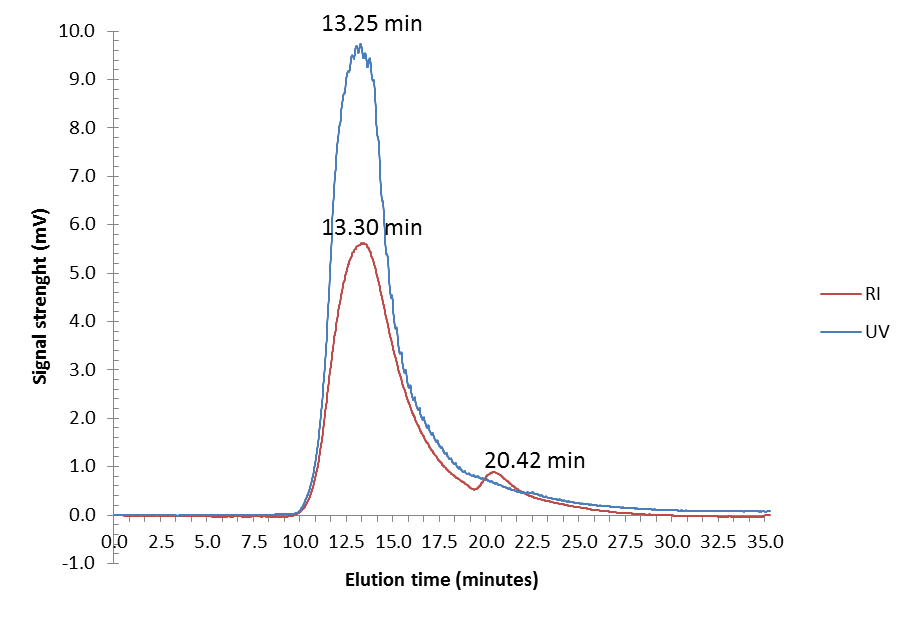


Additional figure 3. *Sc*CBHI Peak 1 HPSEC graph.


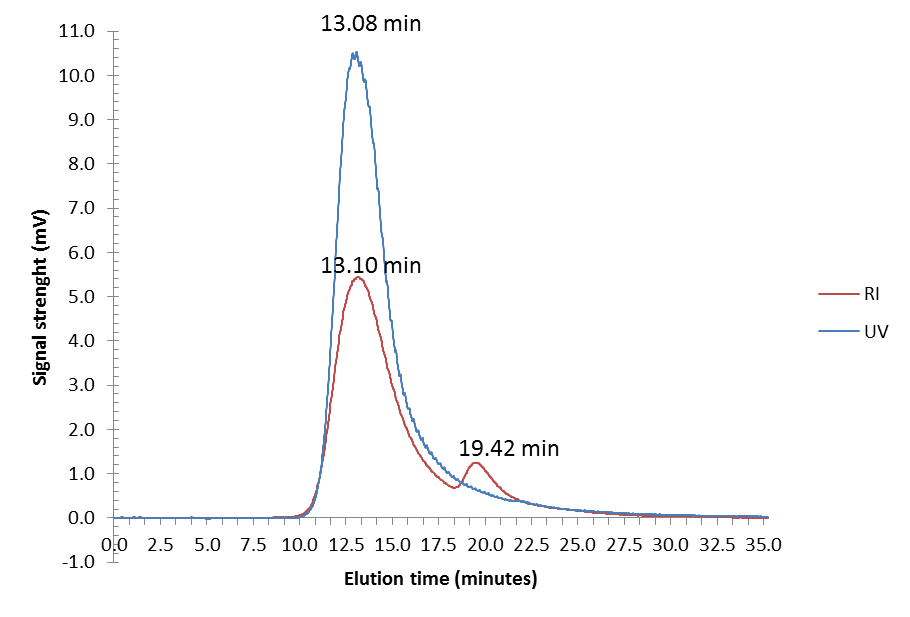


Additional figure 4. *Sc*CBHI Peak 2A HPSEC graph.


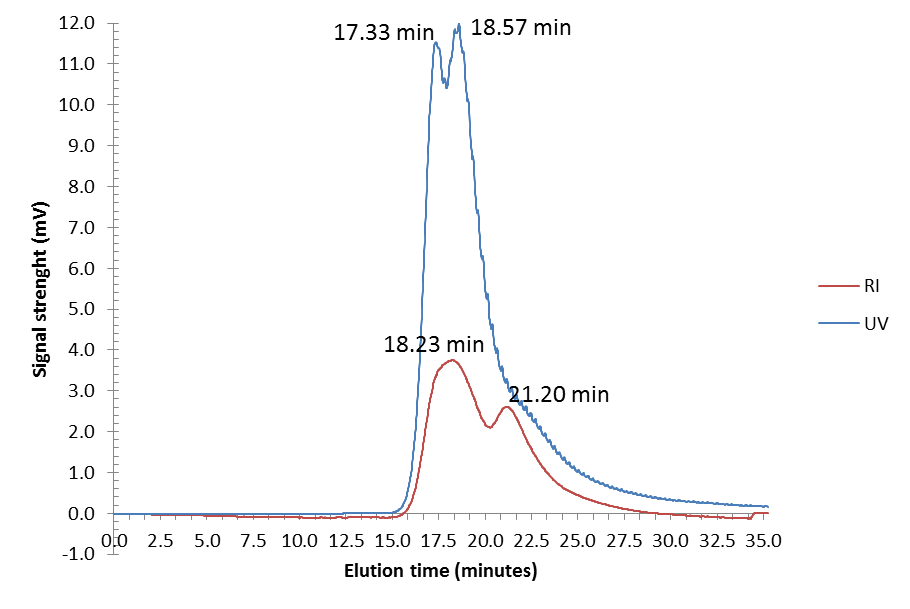


Additional figure 5. *Sc*CBHI Peak 2B HPSEC graph.


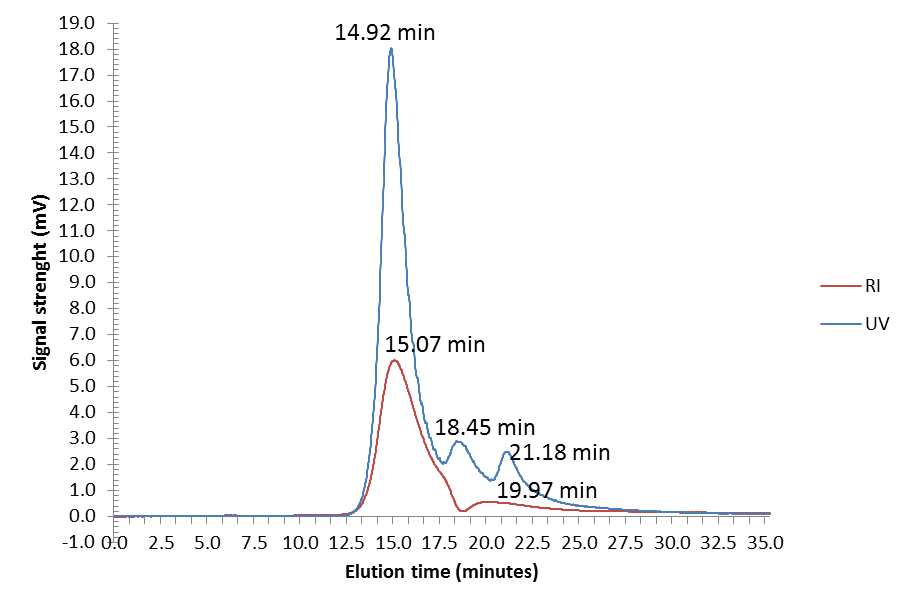


Additional figure 6. *Yl*CBHI HPLC graph.


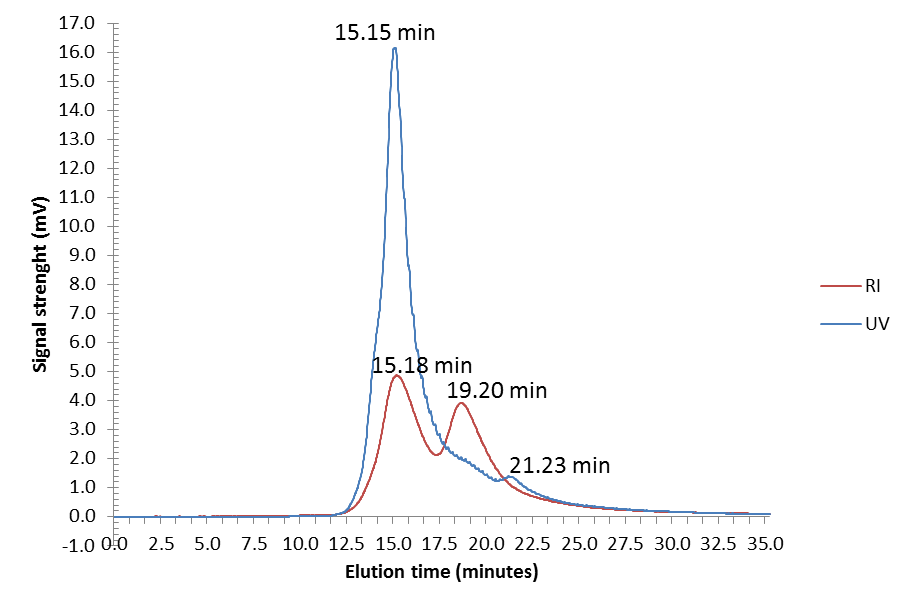


Additional figure 7. *Ls*CBHI HPSEC graph.
